# Distribution of Plasmids in Distinct *Leptospira* Pathogenic Species

**DOI:** 10.1371/journal.pntd.0004220

**Published:** 2015-11-10

**Authors:** Yanzhuo Wang, Xuran Zhuang, Yi Zhong, Cuicai Zhang, Yan Zhang, Lingbing Zeng, Yongzhang Zhu, Ping He, Ke Dong, Utpal Pal, Xiaokui Guo, Jinhong Qin

**Affiliations:** 1 Department of Microbiology and Immunology, Institutes of Medical Science, Shanghai Jiao Tong University School of Medicine, Shanghai, China; 2 Computational Biology Department, Memorial Sloan Kettering Cancer Center, New York, New York, United States of America; 3 National Institute for Communicable Disease Control and Prevention, Chinese Center for Disease Control and Prevention (ICDC, CCDC), Beijing, China; 4 The First Affiliated Hospital of Nanchang University, Nanchang, China; 5 Department of Veterinary Medicine, University of Maryland, College Park and Virginia-Maryland Regional College of Veterinary Medicine, College Park, Maryland, United States of America; Institut Pasteur, FRANCE

## Abstract

Leptospirosis, caused by pathogenic *Leptospira*, is a worldwide zoonotic infection. The genus *Leptospira* includes at least 21 species clustered into three groups—pathogens, non-pathogens, and intermediates—based on 16S rRNA phylogeny. Research on *Leptospira* is difficult due to slow growth and poor transformability of the pathogens. Recent identification of extrachromosomal elements besides the two chromosomes in *L*. *interrogans* has provided new insight into genome complexity of the genus *Leptospira*. The large size, low copy number, and high similarity of the sequence of these extrachromosomal elements with the chromosomes present challenges in isolating and detecting them without careful genome assembly. In this study, two extrachromosomal elements were identified in *L*. *borgpetersenii* serovar Ballum strain 56604 through whole genome assembly combined with S1 nuclease digestion following pulsed-field gel electrophoresis (S1-PFGE) analysis. Further, extrachromosomal elements in additional 15 Chinese epidemic strains of *Leptospira*, comprising *L*. *borgpetersenii*, *L*. *weilii*, and *L*. *interrogans*, were successfully separated and identified, independent of genome sequence data. Southern blot hybridization with extrachromosomal element-specific probes, designated as lcp1, lcp2 and lcp3-*rep*, further confirmed their occurrences as extrachromosomal elements. In total, 24 plasmids were detected in 13 out of 15 tested strains, among which 11 can hybridize with the lcp1-*rep* probe and 11 with the lcp2-*rep* probe, whereas two can hybridize with the lcp3-*rep* probe. None of them are likely to be species-specific. Blastp search of the lcp1, lcp2, and lcp3-*rep* genes with a nonredundant protein database of *Leptospira* species genomes showed that their homologous sequences are widely distributed among clades of pathogens but not non-pathogens or intermediates. These results suggest that the plasmids are widely distributed in *Leptospira* species, and further elucidation of their biological significance might contribute to our understanding of biology and infectivity of pathogenic spirochetes.

## Author Summary

Leptospirosis, caused by a diversity of pathogenic species within the genus *Leptospira*, is a worldwide public health problem affecting both developed and developing countries. In 2003, the whole genome sequencing of pathogenic *Leptospira interrogans* strain 56601 opened the genomic research of this specific pathogen and accelerated genomic sequencing speed afterwards. The availability of whole genome sequences of the pathogenic species *L*. *interrogans* and *L*. *borgpetersenii*, saprophyte *L*. *biflexa* and the intermediate *L*. *licerasiae*, which represented three phylogenetic groups of *Leptospira* spp., has undoubtedly facilitated the understanding of the genetic complexity of this organism. Genome drafts of more than 300 strains have been released recently. The analysis of these sequences will provide invaluable information for understanding the evolution and adaption to various environment of the *Leptospiraceae*. Extrachromosomal replicons are important for communication of genetic information between bacteria. Despite first leptospiral genome was sequenced in 2003, these small auto-replicons, however, were not identified in the whole genome sequence of pathogenic *L*. *interrogans* until 2014. How to effectively identify the presence of small auto-replicons in *Leptospira* cell is a technical challenge which hinders researchers in fully understanding the complexity and diversity of *Leptospira* genomes. In this study, we report identification of small auto-replicons in 15 *Leptospira* strains endemic in China by S1-PFGE analysis, independent of genome sequence assembly. Further analysis suggested that these plasmids are widely presented in *Leptospira* species. The study, combined with the recently available genomic sequencing data, will help us to elucidate the diversity of *Leptospira* genomes and a deeper insight into evolutionary perspective of this unique clade of organism.

## Introduction

Leptospires are thin, spiral, highly motile bacteria that belong to the order Spirochaetales, an early branch of eubacteria. The genus *Leptospira* includes at least 21 species based on 16S rRNA phylogeny, further distinguished into three clades: pathogens, non-pathogens and intermediates[1]. Pathogenic *Leptospira* are comprised of at least 14 species, which share a common branch in evolution, genetically distinct from non-pathogens. Leptospires are also serologically classified into serovars, including more than two hundred that are pathogenic in human and animals[1].

Pathogenic *Leptospira* is known to cause the widespread water-related zoonosis, called leptospirosis. Hosts usually become infected through direct contact with soil or water contaminated by the urine of infected animals [2]. Infection produces a wide spectrum of clinical manifestations, ranging in severity from a mild influenza-like disease to an acute, potentially lethal infection. Pathogenic *Leptospira* species, such as *L*. *interrogans*, *L*. *borgpetersenii*, *L*. *kirschneri*, *L*. *noguchii*, and *L*.*weillii* [3–5], are the causative pathogens of leptospirosis, among which *L*. *interrogans* and *L*. *borgpetersenii* are most prevalent globally. Although infection with *L*. *interrogans* and *L*. *borgpetersenii* cause similar clinical symptoms, the transmission modes are different: for example, *L*. *interrogans* is commonly waterborne, whereas *L*. *borgpetersenii* is transmitted via direct host-to-host contact [6]. Currently, the completed genome sequences include five pathogenic strains of *L*. *interrogans*, two pathogenic strains of *L*. *borgpetersennii*, and two strains of saprophytic *L*. *biflexa* [6–12]. Comparison of the *L*. *interrogans* and *L*. *borgpetersenii* genome sequences revealed genome reduction in the *L*. *borgpetersenii* genome that is supposedly IS-mediated [6].

Plasmids are regarded as one of the most effective vehicles for bacterial communication of genetic information [13], promoting the rapid evolution and adaptation abilities of bacteria [14]. Because of the diverse genetic information they carry, plasmids often play specific biological roles in the host bacterium. Also, they can potentially be engineered as efficient genetic tools for microbial genetic manipulation and analysis through the introduction, modification, or removal of target genes [15]. In fact, many plasmids were found in spirochaetes, with a majority identified in the genus *Borrelia [13].* In addition to its linear chromosome, *Borrelia* contains multiple circular and linear plasmids within a single cell [16]. As for the genus *Leptospira*, plasmid P74 and bacteriophage LE1 were first reported within the saprophytic *L*. *biflexa* serovar Patoc strain Patoc I genome, and then a LE1-like prophage was found in the intermediate species *L*. *licerasiae* [9,17]. It was long believed that pathogenic *Leptospira* species contain only two chromosomes [5]. Recently, a 54-kb genomic island, LaiGI, was confirmed as an extrachromosomal replicon stable within the first sequenced pathogenic strain of *L*. *interrogans* strain Lai [18,19]. Two plasmids, pGui1 and pGui2, in *L*. *interrogans* pathogenic strain Gui44 and three plasmids, lcp1, lcp2, and lcp3, in *L*. *interrogans* pathogenic strain 56609 were reported in succession, which significantly contribute to revealing the diversity of the pathogenic *Leptospira* genome[10,12]. Based on the plasmids identified, *L*. *interrogans–Escherichia coli* shuttle vectors with the predicted replication *rep* gene or *rep* combined with *parAB* loci from the three plasmids of *L*. *interrogans* pathogenic strain 56609 were reported to be successfully transformed into both saprophytic and pathogenic *Leptospira* species, which is considered as a new milestone in research efforts involving pathogenic *Leptospira* [12,16].

Although the plasmids have originally been identified in *L*. *interrogans*, the plasmid sequence in other infectious strains, including *L*. *borgpetersenii* is still unknown. In this study, two extrachromosomal circular elements of *L*. *borgpetersenii* serovar Ballum strain 56604 were detected and estimated by S1 nuclease digestion following pulsed-field gel electrophoresis (S1-PFGE), which allowed detection of low-copy replicons [20]. These two plasmids were further characterized in details through whole genome sequencing. We subsequently used S1-PFGE to identify the plasmids in the remaining 14 reference strains of *Leptospira* in China belonging to species *L*. *borgpetersenii*, *L*. *interrogans*, and *L*. *weilii*. These efforts will contribute to a better understanding the genetic complexity of this bacterium, delivering the most effective genomic information, and accelerating the process of complete genomic sequencing of the genus *Leptospira*.

## Materials and Methods

### Bacterial strains and culture conditions

*Leptospira borgpetersenii* serogroup Ballum serovar Ballum strain 56604 was obtained from the Institute for Infectious Disease Control and Prevention (CDC), Beijing, China. The remaining 14 domestic reference strains of *Leptospira* were also obtained from the Chinese CDC and used in this study: *Leptospira interrogans* serogroup Icterohaemorrhagiae serovar Lai strain 56601, serogroup Canicola serovar Canicola strain 56603, serogroup Pyrogenes serovar Pyrogenes strain 56605, serogroup Autumnalis serovar Autumnalis strain 56606a and 56606v, serogroup Australis serovar Australis strain 56607, serogroup Pomona serovar Pomona strain 56608, serogroup Grippotyphosa serovar Linhai strain 56609, serogroup Hebdomadis serovar Hebdomadis strain 56610, serogroup Bataviae serovar Paidjan strain 56612, serogroup Sejroe serovar Wolffi strain 56635; *Leptospira borgpetersenii* serogroup Javanica serovar Javanica strain 56602, serogroup Tarassovi serovar Tarassovi strain 56613, serogroup Mini serovar Mini strain 56655; *Leptospira weilii* serogroup Manhao serovar Qingshui strain 56615. Of note, 56606a is avirulent strain derived from strain 56606v and has lost its virulence after long time *in vitro* passages in our laboratory. The strains were grown in liquid Ellinghausen–McCullough–Johnson–Harris (EMJH) medium under aerobic conditions at 28°C to mid-log phase and then collected at an optical density of 1.3–2.0 at 600 nm.

### Next-generation DNA sequencing and assembly

The genome of *L*. *borgpetersenii* serovar Ballum strain 56604 was sequenced using a 454 GS20 system (454 Life Sciences). Six micrograms of 56604 genomic DNA was prepared to create sequencing libraries according to the manufacturer’s protocol (454 Life Sciences). A total of 421,952 reads (average read length 282bp) were generated and 421,410 reads of high quality (99.8%) were selected for genome assembly, providing 29.5 fold coverage. 179 contigs (144 contigs >500bp) were yielded and the N50 size of the contigs was 28,039bp. In the finishing process, the reference genome sequences of *L*. *borgpetersenii* serovar Hardjo strain L550 and JB197 were used to determine the suppositional contig order of 56604. In the following physical gap closing, PCR was performed thousands of times based on the following conditions: 5 min at 95°C, followed by 35 cycles of 30 sec at 95°C, 30 sec at 58°C, and 30 sec at 72°C. After that, sequence assembly was accomplished using Phred, Phrap, and Consed programs[21–23].

### Genome annotation and sequence similarity analysis

The sequence alignments were performed using BLAST (http://blast.st-va.ncbi.nlm.nih.gov/Blast.cgi). The open reading frames (ORFs) were predicted and manually checked by the combined use of GLIMMER, GeneMark, and Z-curve programs[24,25]. Clusters of orthologous groups (COG) functional annotation for each gene was performed through RPS-BLAST in the NCBI Conserved Domain Database (CDD), and conserved domains were analyzed to further verify and supplement the annotation by searching the Pfam database[26,27]. Transfer RNA genes were identified with tRNAscan-SE (http://selab.janelia.org/tRNAscan-SE/). Insertion sequence (IS) elements were determined using the IS-finder online tool (https://www-is.biotoul.fr/). Orthologous proteins were identified by performing BLAST searches against the NCBI non-redundant protein database of *L*. *borgpetersenii* serovar Hardjo strain L550 and strain JB197 genomes and *L*. *borgpetersenii* serogroup Ballum serovar Ballum strain 56604 and subsequently checked manually. Whole-genome sequence comparison was performed at the nucleotide level using the program BLASTn (with a cutoff E value of 1e-10) and visualized with EasyFig[28].

### Preparation of agarose gel plugs

Bacteria were grown in EMJH medium under aerobic conditions at 28°C to mid-log phase. Each culture was suspended in 100 μl cell suspension buffer (50mM Tris-HCl pH7.2, 25M NaCl, 50mM EDTA) to a turbidity of 1.3–2.0 OD_600_, as specified in S1 Table. An equal volume of plasmid-harboring bacteria was embedded in agarose plugs (1%) (SeaKemGold Agarose gels), immediately dispensed into wells of plug molds, then incubated at 55°C with proteinase K solution (2% (w/v) Na-deoxycholate, 10% (w/v) Na-lauroyl sarcosine, 0.5M EDTA pH 8.0, 0.5 M NaCl, 0.5% proteinase K) overnight. The plugs were washed twice in tubes shaking in a 54°C water bath for 15 min each time with both pre-warmed double distilled water and TE buffer to inactivate the proteinase K, then used immediately or stored in TE buffer at 4°C.

### S1 nuclease and restriction enzyme digestion of DNA in plugs and pulsed-field gel electrophoresis (PFGE)

S1 nuclease, a specific enzyme that can convert supercoiled plasmids into full-length linear molecules, was used to digest the 2-mm gel plugs. For different *Leptospira* species, the digestion reaction was similar in time but different in the enzyme added (S1 nuclease, Thermo Scientific; *Not*I and *Pst*I, New England Biolabs), as shown in S1 Table. The standard strain *Salmonella enterica* serotype Braenderup (H9812), kindly provided by the Department of Clinical Microbiology, Ruijin Hospital (Shanghai, China), was digested with *Xba*I and used as a molecular weight marker. Gel plugs were subjected to PFGE immediately after completion of the digestion reaction using a contour-clamped homogeneous electric field machine (CHEF-DR III; Bio-Rad). Electrophoresis with linear ramp time from 5 to 65 s at a gradient of 6 V/cm and an included angle of 120° was performed for 20 h to separate the DNA fragments, and gels were cooled continuously at 14°C during the running process. Gels were then stained in 1μg/mL ethidium bromide for 40 min and visualized in a gel image acquisition and analysis system.

### Preparation of probe and southern hybridization

The plasmid DNA was transferred and cross-linked to positively charged nylon membranes (Roche Diagnostics) and hybridized against digoxigenin-labeled probes generated through polymerase chain reaction amplification (PCR DIG Probe Synthesis Kit, Roche) according to the manufacturer’s instructions with some modifications. Briefly, the membrane was washed in 2X SSC twice for 5 min each time at 25°C, then washed twice in 0.1X SSC for 15 min at 68°C. After blocking in 1% blocking reagent for 30 min, the membrane with DIG-labeled probe was detected with anti-Digoxigenin-AP, Fab fragments (Roche), and CDP-*Star* (Roche). All primers used are listed in S2 Table.

### Nucleotide sequence accession number

The complete genomic sequences of *L*. *borgpetersenii* strain 56604 have been deposited in GenBank under the following accession numbers CP012029, CP012030, CP012031 and CP012032.

## Results

### General features of *L*. *borgpetersenii* strain 56604

The genome of strain *L*. *borgpetersenii* strain 56604 consists of two circular chromosomes and two circular extrachromosomal replicons. Chromosome CI and CII are 3,550,837 bp and 361,762 bp, respectively, with GC content of 40.2%. Table 1 summarizes the genome features of strains 56604, L550 and JB197. The genome of strain 56604 is smaller than *L*. *interrogans*, similar to that of *L*. *borgpetersenii* strains L550 and JB197[6].

**Table 1:** General features of the *L*. *borgpetersenii* serovar Ballum and serovar Hardjo genome.

Genome Features	Ballum 56604				Hardjo-bovis L550		Hardjo-bovis JB197	
	CI	CII	lbp1	lbp2	CI	CII	CI	CII
**Genome Size(bp)**	3.55Mb	362Kb	65Kb	59Kb	3.61Mb	317Kb	3.58Mb	300Kb
**G+C content (%)**	40.2	40.2	41	39.7	40.23	40.16	40.23	40.43
**Protein coding (%)**	75.94	75.95	75.81	75.5	74	73.9	73.8	74.8
**Total CDSs**	2,360	258	39	35	2,607	235	2,540	230
**CDSs with assigned function**	1,828	196	26	24	1,647	134	1,594	131
**CDSs without assigned function**	532	62	13	11	960	101	946	99
**Average CDS length(bp)**	1,142	1,065	1,272	1,285	1,026	998	1,039	975
**Total IS element**	45	8	1	0	114	7	111	5
**IS1533**	26	5	0	0	72	5	81	3
**Transfer RNA**	37	0	1	0	37	0	35	0
**23S Ribosomal RNA**	2	0	0	0	2	0	2	0
**16S Ribosomal RNA**	2	0	0	0	2	0	2	0
**5S Ribosomal RNA**	1	0	0	0	1	0	1	0

**Fig 1:**
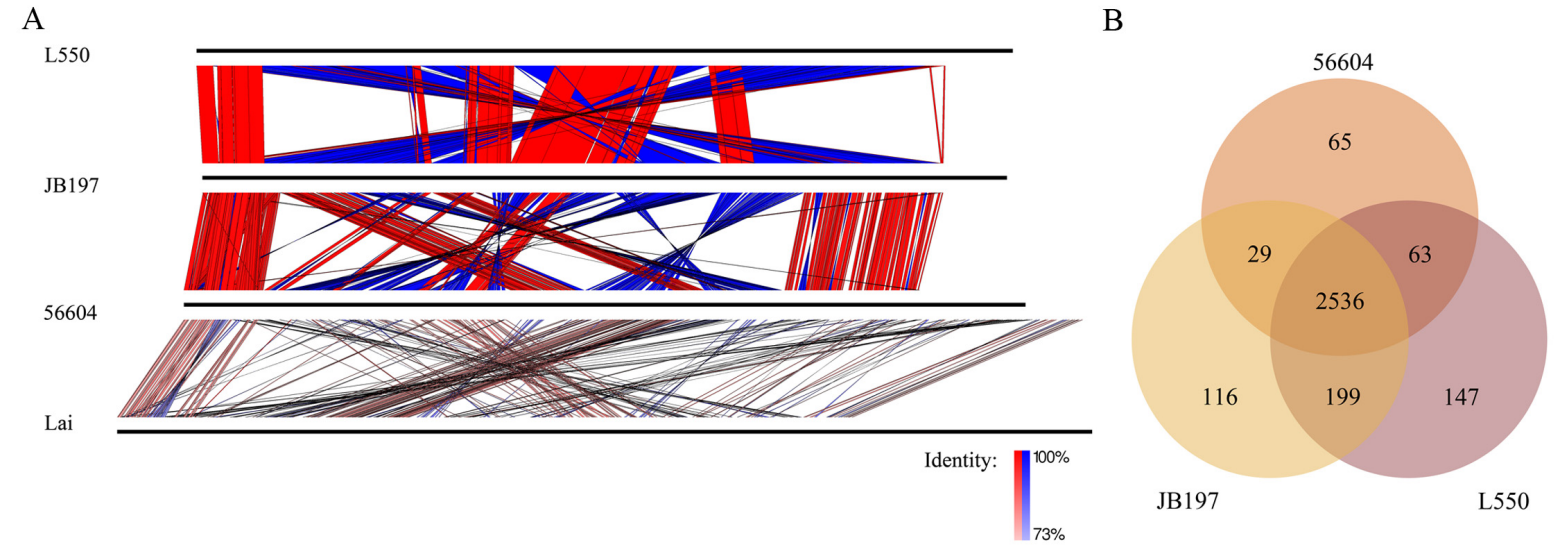
Comparison of replicons and the shared and unique orthologs of *L*. *borgpetersenii*. **(**A) The replicons of *L*. *borgpetersenii* serovar Hardjo strains L550 and JB197, serovar Ballum strains 56604 and *L*. *interrogans* serovars Lai were aligned by BLASTn (with a cutoff E value of 1e-10) and visualized with EasyFig[28]. Colored lines drawn between two adjacent linearized chromosomes (horizontal black lines) showed the location of homologous regions and vertical blocks between sequences indicated regions of shared similarity in the same (red) or opposite (blue) direction. (B) Venn diagram showing the distribution of shared and unique proteins by *L*. *borgpetersenii* species (BLASTp with a cutoff E value of 1e-5, identity of ≥50% and coverage of ≥50%).

The genome sequence of strain 56604, L550 and JB197 shared extensive collinear disrupted by few rearrangements, but not *L*. *interrogans* strain Lai (Fig 1A).The numbers of conserved genes amongst the three strains are 2,536 as shown in Fig 1B. Two additional circular extrachromosomal replicons, designated lbp1 and lbp2, which are 65,435 bp with GC content of 41% and 59,545 bp with GC content 39.7%, respectively (detailed in Table 1 and S1 Fig) were present in strain 56604. Several insertion sequence (IS) elements have been described in pathogenic *Leptospira*, including IS1500, IS1501, IS1533, and ISLin1[5,29]. We identified a total of approximately 54 ISs scattered in chromosomes of strain 56604, including 31 copies of IS1533, 15 copies of ISLin1, 4 copies of IS1502, 2 copies of IS1500, 1 copy of IS1501, and 1 copy of ISLin2(S3 Table). The number of IS copies is apparently less than those in *L*. *borgpetersenii* strain L550 and JB197 [6,30,31].

### Two extrachromosomal replicons, designated lbp1 and lbp2, share similar replication sequence with lcp1 and lcp2

Two circular extrachromosomal replicons, designated lbp1 and lbp2, were identified in strain 56604 through whole genome sequencing. According to sequence-reads coverage of each assembly level, the two plasmids were estimated to share equal copy numbers with the chromosomes. The GC content is higher than that of plasmids lcp1, lcp2, and lcp3 (35%, 35%, 39%) in *L*. *interrogans* strain 56609 as well as pGui1 (34.63%) and pGui2 (33.33%) in *L*. *interrogans* strain Gui44 (Table 2)[10,12]. Sequence comparisons showed low similarity amongst them (less than 20% coverage). BLASTp analysis showed that plasmid lbp1 has 1, 3, 0, 10 and 1 orthologs in plasmid lcp1, lcp2, lcp3, pGui1and pGui2 respectively; that plasmid lbp2 has 1, 1, 0, 1 and 3 orthologs in plasmid lcp1, lcp2, lcp3, pGui1 and pGui2 respectively.

**Table 2:** General features of the plasmids in sequenced pathogenic *Leptospira* genome.

Features	*L*. *borgpetersenii* serovar Ballum strain 56604		*L*. *interrogans* serovar Linhai strain 56609			*L*. *interrogans* serovar Canicola strain Gui44	
	lbp1	lbp2	lcp1	lcp2	lcp3	pGui1	pGui2
**Size (bp)**	65,435	59,545	67,282	56,757	54,986	74,981	66,851
**G+C content (%)**	41	39.7	35.91	34.67	39.43	34.63	33.33
**Protein coding (%)**	75.81	75.5	75.4	68.8	83.9	78.32	72.5
**Protein-coding genes**	39	35	71	56	77	62	63
**Hypothetical genes**	13	11	31	30	30	35	52
**Gene density (bp per gene)**	1,272	1,285	714	698	599	947	769
**Transposases**	1	1	6	7	0	9	4
**t RNA**	1	0	0	0	0	0	0
**r RNA**	0	0	0	0	0	0	0

99% identity with 100% coverage with lcp2-*rep*. *parAB* genes were also found to be located immediately upstream of *rep* genes in lbp1 and lbp2 respectively. The *parAB* genes are less conserved and they showed low similarities between each other.

**Fig 2:**
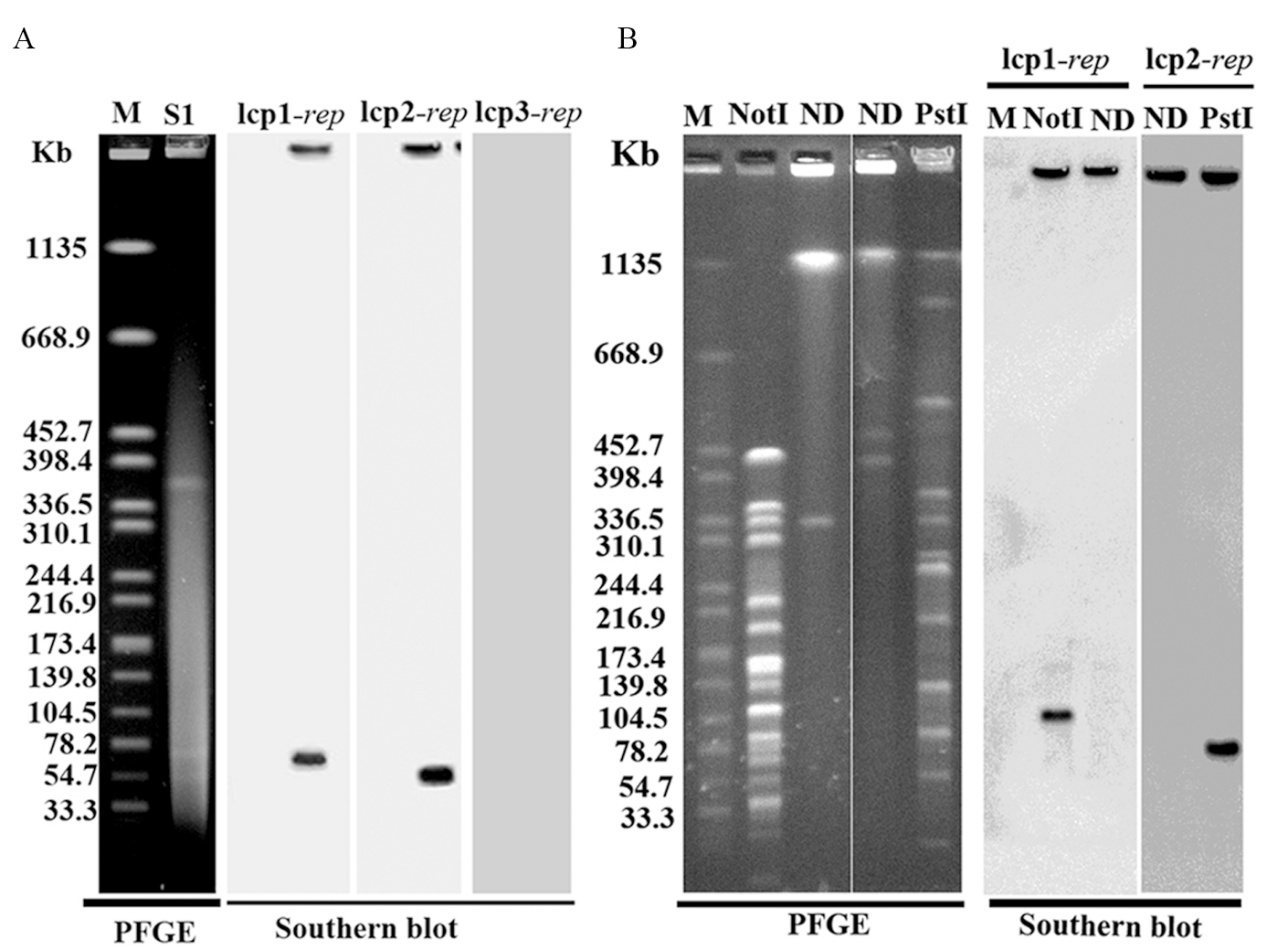
Detection of lbp1 and lbp2 plasmids by PFGE. **(**A) S1-PFGE-based Southern blot analysis. (B) Single restriction enzyme digestion of the plasmids followed by Southern blot analysis. M represents the standard strain *Salmonella enterica* serotype Braenderup (H9812) digested with *Xba*I electrophoresed under pulsed-field conditions as a molecular weight marker. ND, undigested *L*. *borgpetersenii* serovar Ballum strain 56604; *L*. *borgpetersenii* serovar Ballum strain 56604 were digested by S1 nuclease, *Not*I and *Pst*I. For Southern blot analysis, the genomic DNA of *L*. *borgpetersenii* serovar Ballum strain 56604 was blotted to a nylon membrane and hybridized by lcp1-*rep* and lcp2-*rep*. Probes were generated by PCR of T-vector plasmid DNA containing *rep* genes with primer pairs of lcp1-*rep*-probe FR, lcp2-*rep*-probe-FR and lcp3-*rep*-probe-FR, respectively (S2 Table).

S1-PFGE separation followed by Southern blotting with lcp1-*rep*, lcp2-*rep*, and lcp3-*rep* specific probes confirmed that lbp1 could hybridize with lcp1-*rep* sequence and lbp2 with lcp-2-*rep* sequence (Fig 2). Genomic DNA digested with selected restriction enzyme followed by *in situ* PFGE-based Southern blot analysis also confirmed that lbp1 and lbp2 were not integrated into chromosomes. lbp1, cutting with a single restriction enzyme *Not*I in lbp1was confirmed by Southern blotting using lcp1-*rep*. lbp2, cutting with a single restriction enzyme *Pst*I in lbp2 was confirmed by Southern blotting using lcp2-*rep*. The obtained DNA bands were same as above (Fig 2). These data showed that lbp1 contained same *rep* sequence with lcp1 and lbp2 contained same *rep* sequence with lcp2, both of which are extrachromosomal. Further bioinformatics analysis showed that the *rep* of lbp1 shared 87% identity with 100% coverage with lcp1-*rep*, and the *rep* of lbp2 shared

### S1-PFGE detection of plasmids within the 15 Chinese epidemic *Leptospira* strains

**Fig 3:**
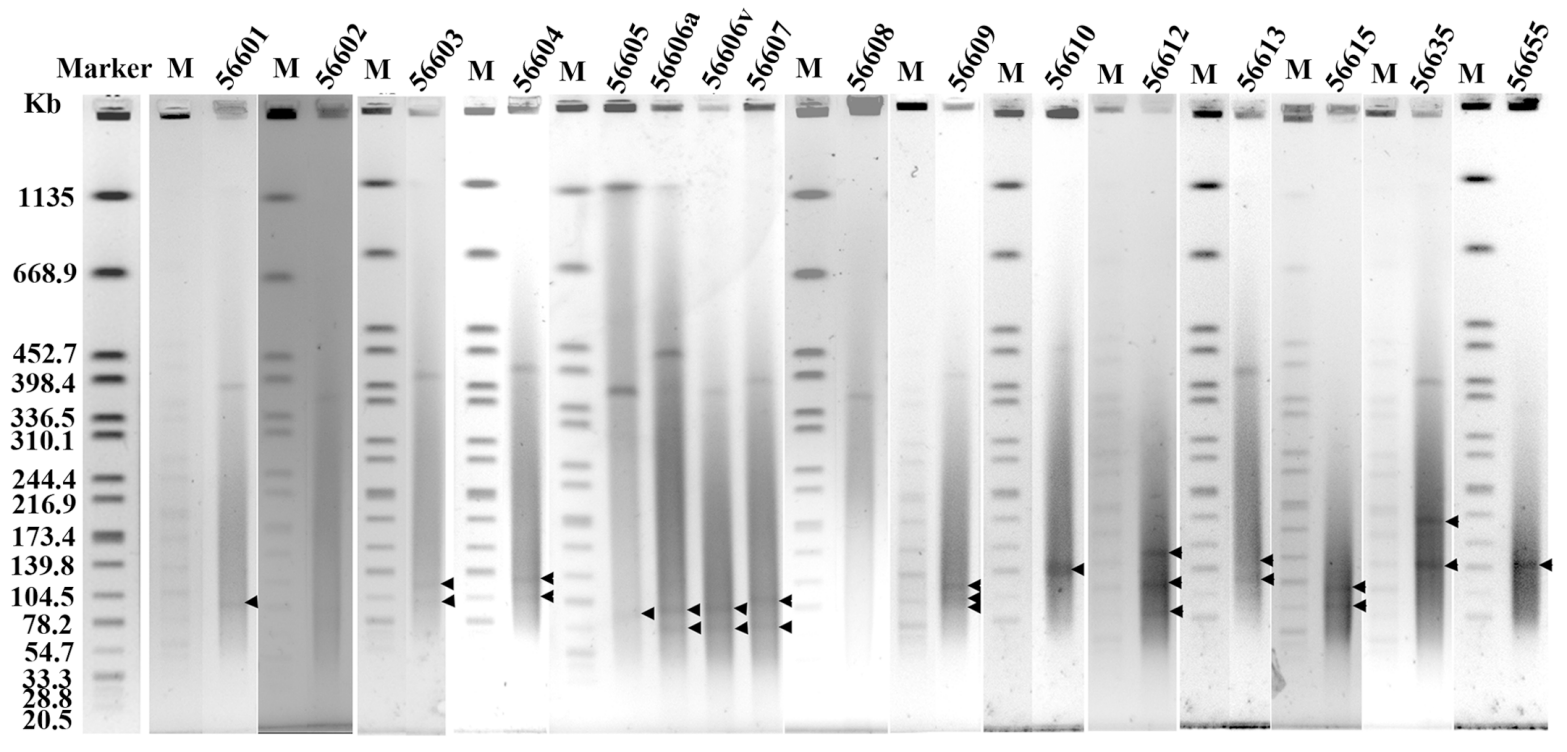
Plasmids detection in 15 Chinese epidemic *Leptospira* strains by S1-PFGE separation. *Leptospira* strains were embedded in agarose, lysed and digested with S1 nuclease and electrophoresed under pulsed-field conditions. Marker represents the pattern of the standard strain *Salmonella enterica* serotype Braenderup (H9812) digested with *Xba*I electrophoresed under pulsed-field conditions as a molecular weight marker. M represents the marker electrophoresed with each sample. 56601–56655 represent 15 Chinese epidemic *Leptospira* strains as detailed in Table 3.

The large size and low copy of plasmids within *Leptospira* cells presents a challenge for their isolation[12]. Moreover, the high proportion of homologous sequence also makes it difficult to differentiate them from the chromosome and affects the assembly process, even during whole genome sequencing[12]. S1 nuclease treatment can convert the supercoiled plasmids into full-length linear molecules. When the bacteria harboring plasmid embedded in agarose are digested with S1 nuclease followed by pulsed-field gel electrophoresis (PFGE), plasmids can be detected and their sizes can be estimated with appropriate linear DNA markers [20]. In this study, 15 Chinese epidemic *Leptospira* strains were subjected to S1-PFGE, a distinct approach to detect the presence and the size of plasmids within *Leptospira* cells. According to the results of S1-PFGE, plasmids can be directly detected in 13 out of 15 tested *Leptospira* strains, with the exception of *L*. *borgpetersenii* strain 56602 and *L*. *interrogans* strain 56608 (Fig 3 and Table 3). The sizes ranged from 50 kb to 150 kb (Fig 3), and one *Leptospira* cell can contain up to three plasmids. It showed that the plasmids have been detected in all tested species, including *L*. *borgpetersenii*, *L*. *weilii*, *and L*. *interrogans*.

**Table 3:** Homology sequence of lcp1-*rep*, lcp2-*rep* and lcp3-*rep* detected in 15 Chinese epidemic *Leptospira* strains.

Strain	Species	Serovar	lcp1-*rep*	lcp2-*rep*	lcp3-*rep*
**56601**	*L*.*interrogans*	Lai	0	1	0
**56602**	*L*.*borgpetersenii*	Javanica	0	0	0
**56603**	*L*.*interrogans*	Canicola	1	1	0
**56604**	*L*.*borgpetersenii*	Ballum	1	1	0
**56605**	*L*.*interrogans*	Pyrogenes	1	0	0
**56606a**	*L*.*interrogans*	Autumnalis	1	1	0
**56606v**	*L*.*interrogans*	Autumnalis	1	1	0
**56607**	*L*.*interrogans*	Australis	1	0	1
**56608**	*L*.*interrogans*	Pomona	0	0	0
**56609**	*L*.*interrogans*	Linhai	1	1	1
**56610**	*L*.*interrogans*	Hebdomadis	0	1	0
**56612**	*L*.*interrogans*	Paidjan	2	1	0
**56613**	*L*.*borgpetersenii*	Tarassovi	1	1	0
**56615**	*L*.*interrogans*	Qingshui	1	1	0
**56635**	*L*.*borgpetersenii*	Wolffi	1	1	0
**56655**	*L*.*weilii*	Mini	0	1	0

Number represents the band detected by Southern blot hybridized by specific *rep* sequence probes following S1-PFGE separation.

### S1-PFGE-based Southern blot analysis of *rep* gene homology among *Leptospira* with lcp1, lcp2, and lcp3-*rep* specific probes

**Fig 4:**
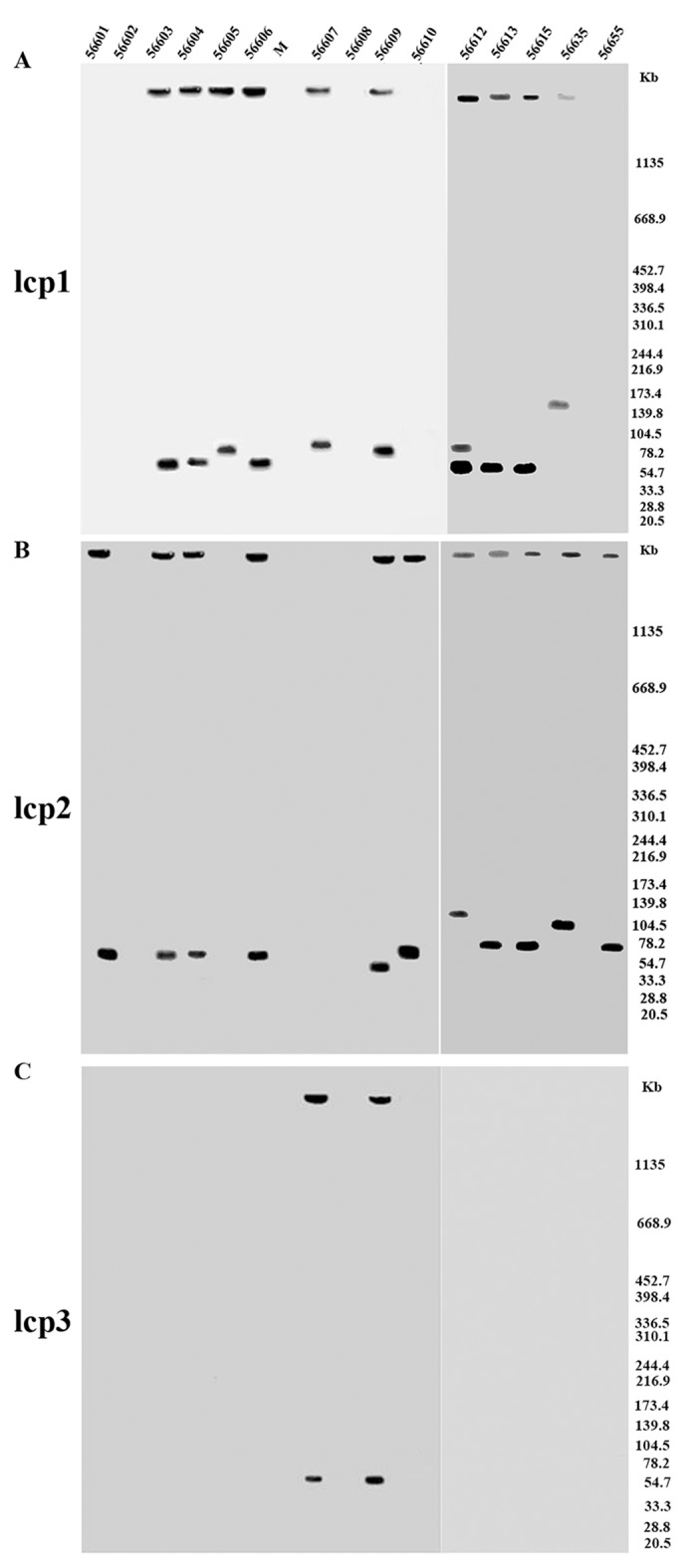
Identification of homologous sequence of lcp1-*rep*, lcp2-*rep* and lcp3-*rep* in 15 Chinese epidemic *Leptospira* strains. *Leptospira* strains were embedded in agarose, lysed and digested with S1 nuclease and electrophoresed under pulsed-field conditions, blotted to a nylon membrane and then hybridized with the probes of lcp1-*rep*(A), lcp2-*rep*(B) and lcp3-*rep*(C). Probes were generated by PCR of T-vector plasmid DNA containing *rep* genes with primer pairs of lcp1-*rep*-probe FR, lcp2-*rep*-probe-FR and lcp3-*rep*-probe-FR, respectively (S2 Table). Markers are shown on the right. 56601–56655 represent 15 *Leptospira* Chinese epidemic strains as detailed in Table 3.

In our previous study, the whole genome of *L*. *interrogans* serovar Linhai strain 56609 was sequenced, and three extrachromosomal replicons, designated lcp1, lcp2, and lcp3, were in the cell[12]. The homology of *rep* genes in the plasmids detected by S1-PFGE was tested by Southern blot hybridization using lcp1, lcp2, and lcp3-*rep* specific probes. As shown in Fig 4 and Table 3, each of the plasmids detected by S1-PFGE in 13 Chinese epidemic strains can hybridize with one of three known *rep* specific probes. Eleven plasmids distributed in 10 strains can hybridize with lcp1-specific probes (Fig 4A). Of note, two plasmids in strain 56612 can hybridize with lcp1-specific probes. Eleven plasmids distributed in 11 strains can hybridize with lcp2-specific probes (Fig 4B). Two plasmids distributed in two strains can hybridize with lcp3-specific probes (Fig 4C). Plasmid partitioning system encoded two partitioning proteins *ParAB* protein and a replication protein *rep* protein. Plasmids with the same replication control are “incompatible”, whereas the plasmids with different replication control are “compatible”[32]. *rep* has been frequently used to classify plasmids[33]. It seems that the plasmids in Chinese epidemic strains can be divided into three types: lcp1, lcp2, and lcp3.

When compared with the previous results to test for presence of the *rep* gene in the same 15 Chinese epidemic strains, more plasmids were detected in this study[12]. The only difference is that in the previous study, the embedded bacterial cells were digested with the selected restriction enzyme, whereas in this study, the cells were digested with S1 nuclease. In the previous study, some plasmids without cleavage sites for the selected restriction enzyme may have gone undetected because of being supercoiled. S1-PFGE is a general method independent of knowing restriction enzyme cleavage sites and thus can detect all the plasmids in a cell[20].

### lcp1, lcp2, and lcp3-*rep* homologous sequence widely distributed in pathogens (Group I) of genus *Leptospira*

The genus *Leptospira* is comprised of 21 species clustered into following groups, pathogen (Group I), intermediate pathogen (Group II), and non-pathogen based on 16S rRNA phylogeny[1]. The infectious group includes at least 14 species, nine species of pathogens, and five species of intermediate pathogens. The genomes of 319 *Leptospira* strains belonging to 20 species have been sequenced and deposited in the GenBank database. Blastp search of the lcp1, lcp2, and lcp3-*rep* genes against the sequenced *Leptospira* genomes showed that 6 species (299 strains sequenced) including 77 strains harbored homologous sequence with lcp1-*rep*, 6 species (299 strains sequenced) including 80 strains harbored homologous sequence with lcp2-*rep*, and 7 species (301 strains sequenced) including 71 strains harbored homologous sequence with lcp3-*rep* (Table 4). All of the 7 species belonged to pathogens (Group I). Of the 319 sequenced strains, 304 strains belonged to pathogens (Group I); only 8 strains belonged to intermediate pathogens, and 7 strains belonged to the non-pathogens group. Obviously, more genome data of intermediate pathogens and non-pathogens are needed to understand the relationship between extrachromosomal replicons and their host range.

**Table 4:** The distribution of lcp1-*rep*, lcp2-*rep* and lcp3-*rep* in sequenced *Leptospira* strains.

Group	Species	Sequenced strain	lcp1-*rep* strain	lcp2-*rep* strain	lcp3-*rep* strain
pathogens	*L*. *alexanderi*	1	-	-	-
	*L*. *weilii*	9	6	7	7
	*L*. *borgpetersenii*	22	10	10	10
	*L*. *santarosai*	27	7	1	7
	*L*. *meyeri*	2	-	-	-
	*L*. *alstonii*	2	-	-	1
	*L*. *interrogans*	206	39	51	34
	*L*. *kirschneri*	26	9	9	9
	*L*. *noguchii*	9	6	2	3
Intermediate group	*L*. *licerasiae*	4	-	-	-
	*L*. *wolffii*	1	-	-	-
	*L*. *fainei*	1	-	-	-
	*L*. *inadai*	1	-	-	-
	*L*. *broomii*	1	-	-	-
non-pathogens	*L*. *idonii*		-	-	-
	*L*. *vanthielii*	1	-	-	-
	*L*. *biflexa*	2	-	-	-
	*L*. *wolbachii*	1	-	-	-
	*L*. *terpstrae*	1	-	-	-
	*L*. *yanagawae*	1	-	-	-
	*L*. *kmetyi*	1	-	-	-

“-” represents zero.

## Discussion

Heterogeneity within the genus *Leptospira* is well confirmed based on recent studies adopting DNA-DNA hybridization, comparative genomics, and whole genome sequencing efforts [2,5,12]. There are currently 21 species within the genus *Leptospira*, 14 of which are supposedly pathogenic. However, based on prevalence and pathogenicity, *L*. *interrogans* and *L*. *borgpetersenii* are the two largest species, containing about half of the known 230 pathogenic serovars, although the latter strain is less well characterized [6]. *L*. *borgpetersenii* genomes are 700 kb smaller than that of *L*. *interrogans* [6,30] and its genome reduction may be IS-mediated, because there are more copy numbers of IS elements in sequenced *L*. *borgpetersenii* serovar Hardjo compared with in *L*. *interrogans* serovars Lai and Copenhageni [6]. IS sequence, as a mobile genetic element, varies between serovars. Several IS elements, including IS1500, IS1501, IS1502, IS1533, and ISLin1, were identified in *Leptospira* [2,5,6,29,30]. Although the genome size of *L*. *borgpetersenii* serovar Ballum strain 56604 showed a similar trend of genome reduction as *L*. *borgpetersenii* serovar Hardjo, the type and copy number of IS elements in serovar Ballum were apparently less than those in serovar Hardjo, with the exception of two single copy extrachromosomal elements.

Plasmids are important genetic vehicles playing an important role in the processes of genomic organization, adaptation, evolution, and virulence for bacterial pathogens [13]. Also, plasmids are very important genetic tools to manipulate and analyze microorganisms through introduction, modification, or removal of target genes [13]. However, in some cases due to their occurrence in low copy numbers and containment of repeat sequences (homologous to chromosomes), the method to detect plasmids in *Leptospira* is primarily dependent on whole genome sequencing and assembly[10,12]. Even using whole genome sequencing, plasmid could be miss annotated and assembled into chromosomes [19]. Based on the plasmids identified, shuttle vectors constructed with the *rep* genes can be successfully transformed into some pathogenic *Leptospira*[12,16]. Although genetic incompatibility is supposed to hinder successful transformation, the precise mechanism is still unknown[12]. Identification of more replicons in different strains will help to understand the mechanism and facilitate the manipulation of genetic tools. In this study, S1-PFGE was applied as an effective method to detect the plasmids within *Leptospira* cells, which yielded accurate bands and sizes of plasmids in all tested *Leptospira* strains, although despite our best efforts bands were smeared to some degree. We speculate that despite S1-PFGE method is sensitive, the low copy number of plasmids in *Leptospira* probably render them more difficult for detection.

We tested the plasmid distribution among 15 Chinese epidemic *Leptospira* strains, including *L*. *borgpetersenii*, *L*. *weilii*, *and L*. *interrogans* using S1-PFGE, which enable us to detect potentially all plasmids in sequenced strains 56601, 56603(Gui44), and 56609. The band numbers (corresponding to one, two, and three bands, respectively) and size values were consistent with previously identified plasmids. Furthermore, Southern blotting using known lcp1, lcp2, and lcp3 specific probes further confirmed them as extrachromosomal elements. Interesting, it seems that plasmids detected in Chinese epidemic strains can be divided into three types: lcp1, lcp2, and lcp3. They can exist together or separately within *Leptospira* cells. Moreover, same type plasmids (lcp1 in strain 56612) can also coexist in one cell.

Further analysis of the plasmid-encoded genes did not reveal any unique gene cluster but confirmed the distribution and diversity of plasmids in the genus *Leptospira*. Blastp search of lcp1, lcp2, and lcp3-*rep* homologous sequence with 319 sequenced *Leptospira* genomes further showed they are widely distributed in pathogens (Group I) of *Leptospira*. Based on the information provided in this study, we speculate that additional assessment of plasmid contents in additional globally prevalent *Leptospira* strains, should shed new light on significance of these intriguing extrachromosomal DNA elements in leptospiral biology and evolution.

## Supporting Information

S1 Fig Genome maps of *L*.*borgpetersenii* strain 56604. Circles are numbered from outer to inner and are designated as follows. (A-B) Large chromosome (CI) and small chromosome (CII), (C-D) Plasmid lbp1 and lbp2. Circles 1 and 2 denote forward and reverse strand genes (colors represent functional categories according to COGs). Circles 3, tRNA genes and rRNA genes. The two inner circles for the chromosomes display GC content and GC skew calculated using a 1,000 bp (CI) / 600 bp (CII) window sliding 500 bp (CI) /300 bp (CII) at a time. The two inner circles for the plasmids display GC content and GC skew calculated using a 1,000 bp window sliding 900 bp at a time.
(TIF)

S1 Table General conditions for different *Leptospira* strains by S1-PFGE. (DOCX)

S2 Table Primers used in this study. (DOCX)

S3 Table IS elements in *L*. *borgpetersenii* Serovar Ballum Strain 56604. (DOCX)
