# Supplementary figures and images for: Distribution of Plasmids in Distinct Leptospira Pathogenic Species
Source: PLoS Negl Trop Dis. 2015 Nov 10;9(11):e0004220. doi: 10.1371/journal.pntd.0004220 (PMC4640553; doi:10.1371/journal.pntd.0004220)

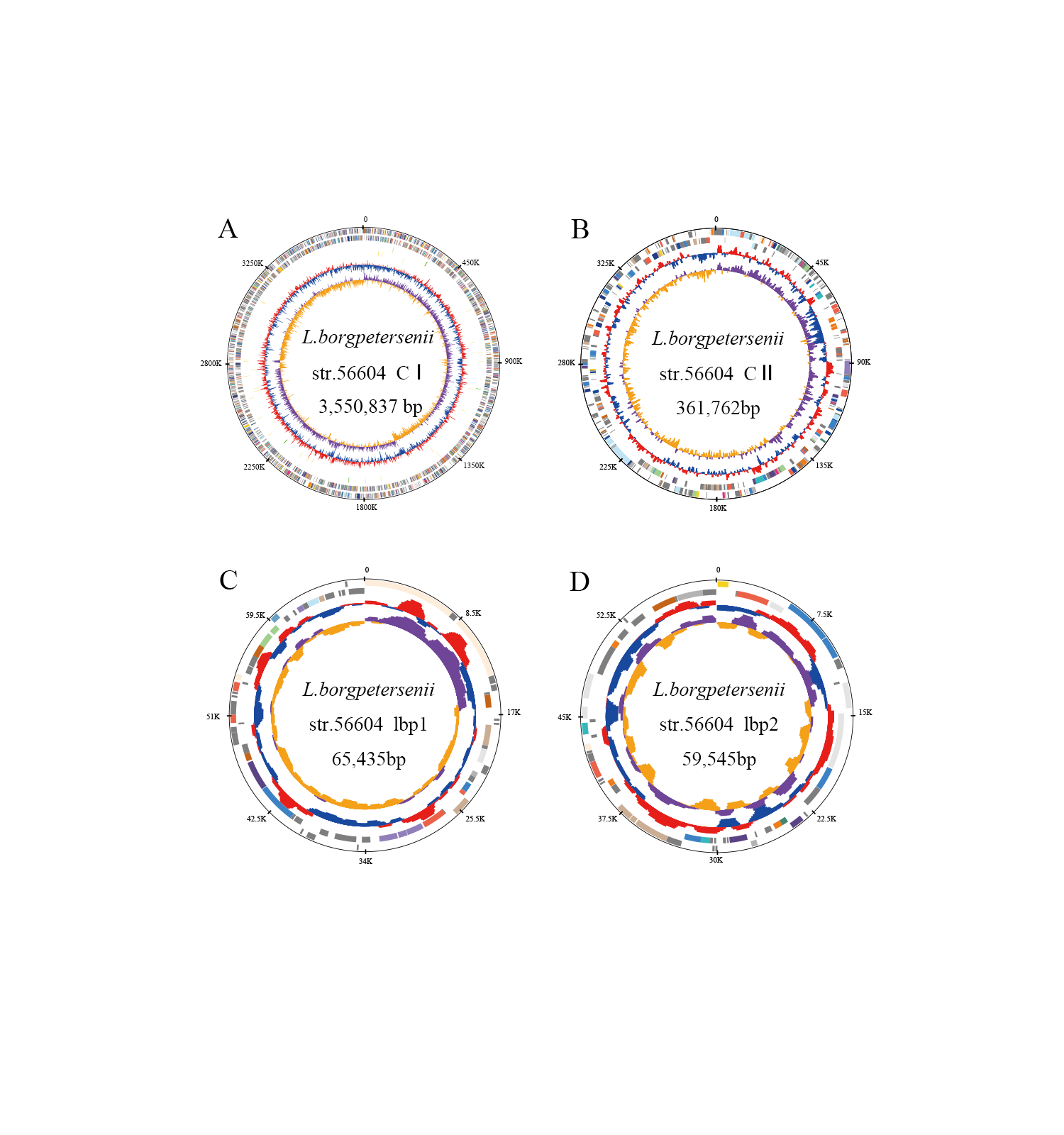

Supplement: S1 Fig — Circles are numbered from outer to inner and are designated as follows. (A-B) Large chromosome (CI) and small chromosome (CII), (C-D) Plasmid lbp1 and lbp2. Circles 1 and 2 denote forward and reverse strand genes (colors represent functional categories according to COGs). Circles 3, tRNA genes and rRNA genes. The two inner circles for the chromosomes display GC content and GC skew calculated using a 1,000 bp (CI) / 600 bp (CII) window sliding 500 bp (CI) /300 bp (CII) at a time. The two inner circles for the plasmids display GC content and GC skew calculated using a 1,000 bp window sliding 900 bp at a time. (TIF) [file pntd.0004220.s001.tif]
