# Supplementary material for: Distribution of Plasmids in Distinct Leptospira Pathogenic Species
Source: PLoS Negl Trop Dis. 2015 Nov 10;9(11):e0004220. doi: 10.1371/journal.pntd.0004220 (PMC4640553; doi:10.1371/journal.pntd.0004220)
Supplement: S1 Table — (DOCX) [file pntd.0004220.s002.docx]

**S1 Table General conditions for different *Leptospira* strains by S1-PFGE**

| strains | Culture concentration(OD_600_) | Enzyme dose(100U/ul) |
| --- | --- | --- |
| 56601 | 2 | 0.2/0.1 |
| 56603 | 1.47 | 0.1 |
| 56604 | 1.6 | 0.1/0.07 |
| 56605 | 2 | 0.1 |
| 56606 | 1.5 | 0.1 |
| 56607 | 2 | 0.1 |
| 56609 | 1.3 | 0.1 |
| 56610 | 1.55 | 0.1 |
| 56612 | 1.6 | 0.2 |
| 56613 | 1.6 | 0.1 |
| 56615 | 1.95/1.3 | 0.1 |
| 56635 | 1.8 | 0.2 |
| 56655 | 1.3 | 0.1 |
