# Supplementary material for: Distribution of Plasmids in Distinct Leptospira Pathogenic Species
Source: PLoS Negl Trop Dis. 2015 Nov 10;9(11):e0004220. doi: 10.1371/journal.pntd.0004220 (PMC4640553; doi:10.1371/journal.pntd.0004220)
Supplement: S2 Table — (DOCX) [file pntd.0004220.s003.docx]

**S2 Table Primers used in this study**

| **Probe** | **Primer ID** | **Primer sequence (5'-3')** | **Product size (bp)** | **Location** |
| --- | --- | --- | --- | --- |
| lcp1-rep | lcp1-rep-probe-F | GTCGTACATTACGCTTTGGT | 771 | 1,845-1,864 (Lcp1) |
| lcp1-rep | lcp1-rep-probe-R | CTTTACTCCCCACTTGTGTGA |  | 2,596-2,616 (Lcp1) |
| lcp2-rep | lcp2-rep-probe-F | GGAAGGTATCTATCTACGGA | 477 | 2,027-2,046 (Lcp2) |
| lcp2-rep | lcp2-rep-probe-R | CTGTACAGTAAACCCCACT |  | 2,486-2,504(Lcp2) |
| lcp3-rep | lcp3-rep-probe-F | GTAGGCGAGTTTATACCTCGT | 601 | 1,829-1,849(Lcp3) |
| lcp3-rep | lcp3-rep-probe-R | CTCAGAGATTTAGAGACTGGA |  | 2,410-2,430(Lcp3) |
