# Supplementary material for: Distribution of Plasmids in Distinct Leptospira Pathogenic Species
Source: PLoS Negl Trop Dis. 2015 Nov 10;9(11):e0004220. doi: 10.1371/journal.pntd.0004220 (PMC4640553; doi:10.1371/journal.pntd.0004220)
Supplement: S3 Table — (DOCX) [file pntd.0004220.s004.docx]

**S3 Table. IS elements in *L. borgpetersenii* Serovar Ballum Strain 56604**

| **Features** | **CⅠ** | **CⅡ** | **lbp1** | **lbp2** |
| --- | --- | --- | --- | --- |
| **Genome Size(bp)** | 3,550,837 | 361,762 | 65435 | 59545 |
| **G+C content (%)** | 40.2 | 40.2 | 41 | 39.7 |
| **Protein coding (%)** | 75.94 | 75.95 | 75.81 | 75.5 |
| **Total CDSs** | 2360 | 258 | 39 | 35 |
| **CDSs with assigned function** | 1828 | 196 | 26 | 24 |
| **CDSs without assigned function** | 532 | 62 | 13 | 11 |
| **Average CDS length(bp)** | 1142 | 1065 | 1272 | 1285 |
| **CDS on forward strand** | 1210 | 222 | 13 | 15 |
| **CDS on reverse strand** | 1150 | 180 | 26 | 20 |
| **Total IS element** | 45 | 8 | 1 | 0 |
| **ISLin1** | 11 | 3 | 1 | 0 |
| **ISLin2** | 1 | 0 | 0 | 0 |
| **IS1500** | 2 | 0 | 0 | 0 |
| **IS1501** | 1 | 0 | 0 | 0 |
| **IS1502** | 4 | 0 | 0 | 0 |
| **IS1533** | 26 | 5 | 0 | 0 |
| **Transfer RNA** | 37 | 0 | 1 | 0 |
| **Ribosomal RNA** | 5 | 0 | 0 | 0 |
